# Recurrent Primary Abdominal Wall Abscess: A Case Report and Review of the Literature

**DOI:** 10.7759/cureus.41069

**Published:** 2023-06-28

**Authors:** Masahiro Shiihara, Yasuhiro Sudo, Norimasa Matsushita, Harushi Osugi, Tatsuo Inoue

**Affiliations:** 1 Gastrointestinal Surgery, Kamifukuoka General Hospital, Saitama, JPN

**Keywords:** abscess, case report, surgical drainage, rectus abdominis, primary abdominal wall abscess

## Abstract

Primary abdominal wall abscess is extremely rare and difficult to diagnose because abdominal wall abscesses usually occur secondary to malignant tumors or inflammatory diseases. We experienced a case of an 80-year-old man with an asynchronous primary abdominal wall abscess with recurrence. Both abscesses were successfully treated with surgical drainage. A patient without any history of cancer or trauma presented to our department with right upper abdominal pain. His laboratory data showed an abnormal high inflammatory response, and computed tomography revealed a 40 × 30 mm mass formed in the rectus abdominis muscle of the upper right abdomen. The mass had no continuity with the surgical scar after cholecystectomy or intra-abdominal organs. *Citrobacter diversus* was detected in the culture from the mass and any epithelial components were not detected by biopsy. For the diagnosis of primary abdominal wall abscess, the patient underwent surgical drainage because antibiotic treatment was ineffective. The abscess disappeared promptly after the drainage. Thirteen months after the first treatment, another primary abdominal wall abscess was noted in the lower right abdomen. The abscess also promptly disappeared with surgical drainage. Primary abdominal wall abscess is difficult to diagnose because of its rarity. Prompt diagnosis and drainage are important to prevent exacerbation.

## Introduction

Most abdominal wall abscesses occur secondary to malignant tumors, inflammatory diseases, surgical site infections, and so on [1,2]. Solitary abdominal wall abscesses that are not secondary infections, in other words, primary abdominal wall abscesses (PAWAs), are extremely rare. Few cases have been described in the literature [3-7]. Because of its rarity, PAWA is difficult to diagnose, which delays appropriate treatment. PAWA was treated by antibiotics, surgical drainage, or laparotomy in the past reports. But it is controversial which is most effective due to its rarity. Patients with PAWA often have many comorbidities and compromised immune systems, thus their treatment is sometimes complicated [1,2].

Here, we present a case of asynchronous multiple recurrent PAWA, which was successfully managed through surgical drainage. The first drainage was delayed due to the diagnostic difficulty, but the second drainage was performed immediately and the patient recovered sooner. Additionally, we reviewed cases in the literature to discuss the clinical features of this rare condition.

## Case presentation

**Figure 1:**
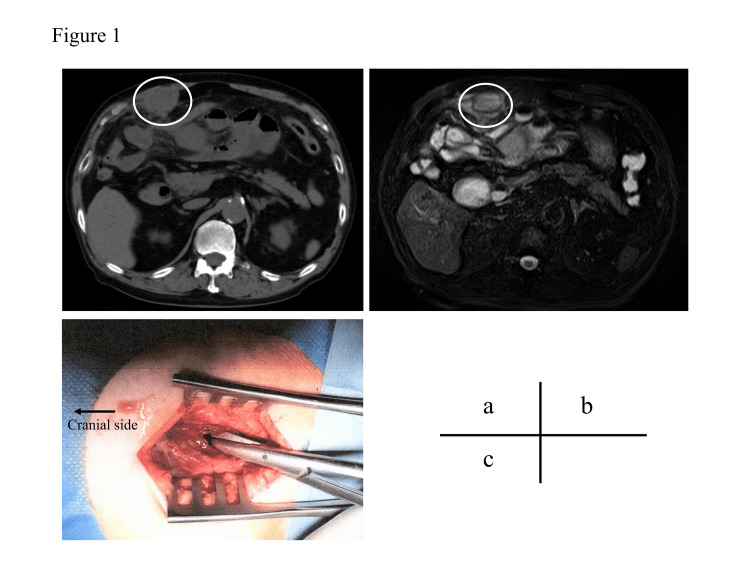
First primary abdominal wall abscess (A) Computed tomography revealed a 40 × 30 mm low-density mass at the right upper abdominal wall. The mass had no connection with any intraperitoneal organs or lymph nodes. (B) Magnetic resonance imaging also showed the mass. (C) Findings of surgical drainage. The abscess was located in the rectus abdominis muscle.

An 80-year-old man presented to our department in July 2020 with a complaint of right upper abdominal pain for about a month. He had atrial fibrillation for which he was taking anticoagulant agents, chronic renal failure, and diabetes mellitus controlled by subcutaneous insulin injections; he underwent open cholecystectomy five years ago; and had no history of cancers, bruises, or trauma. He did not take any immunosuppressants or steroids. He had no fever but had slight swelling and redness in the upper right abdomen with tenderness. Laboratory studies showed an abnormal white blood cell count (WBC) of 10,900/μL, neutrophils at 81.9 %, C-reactive protein (CRP) at 25.58 mg/dL, blood urea nitrogen at 68.1 mg/dL, and creatinine at 3.10 mg/dL. Serum tumor markers, including carbohydrate antigen 19-9 (CA19-9) and carcinoembryonic antigen, were within the normal ranges. Differential diagnoses were hematoma, tumor (primary or metastasis), or abscess. Computed tomography (CT) revealed a round 40 × 30 mm mass with fluid collection located below the abdominal wall, followed from the rectus abdominis muscle (Figure 1A). The mass had no connection with the intestinal tract or lymph nodes, and no other malignant tumors were observed. The surgical site for cholecystectomy was separated from the mass.

**Figure 2:**
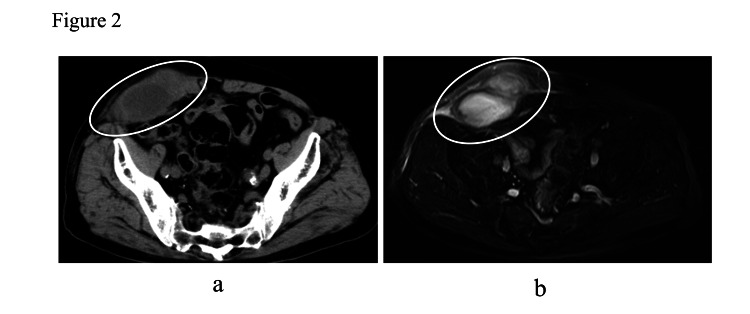
Second primary abdominal wall abscess (A) Computed tomography revealed a 70 × 40 mm low-density mass at the lower abdominal wall, distant from the first one. The mass also had no connection with any intraperitoneal organs or lymph nodes. (B) Magnetic resonance imaging findings.

The patient had tenderness in the same region. On magnetic resonance imaging (MRI), the T2-weighted image showed a high signal, and the diffusion-weighted image showed a low signal mass (Figure 1B). We performed a biopsy and culture of the mass because we could not reach the diagnosis by imaging. A mass biopsy showed no epithelial components or malignant findings, and the diagnosis was an abdominal wall abscess. *Citrobacter koseri* was detected in the culture. None was detected in the blood culture. The patient was hospitalized and treated with antibiotics for over two weeks: tazobactam/piperacillin for a week, cefozopran hydrochloride for a week, and levofloxacin hydrate for five days. However, the abscess did not improve completely, and the laboratory studies showed an abnormal WBC of 10,100/μL and CRP at 10.74 mg/dL. Therefore, surgical drainage was performed 19 days after the first visit. Under local anesthesia, a 5-cm incision was made, and the rectus abdominis muscle was divided to reach the mass. The intra-operative findings showed the abscess cavity located in the rectus abdominis muscle (Figure 1C). The content of the abscess was pus, not including hematoma. A drain was placed postoperatively for a few days. The abscess promptly disappeared after the drainage; however, it took 25 days for discharge due to postoperative aspiration pneumonia, treated with antibiotics. After discharge, the patient did not have regular follow-up visits to our department. In October 2021, the patient was admitted again to our department with right lower abdominal pain with tenderness. Laboratory studies showed an abnormal WBC at 10,300/μL and CRP at 13.49 mg/dL. CT revealed a 70 × 40 mm mass with fluid collection distant from the previous abscess cavity and a high signal on the T2-weighted image on MRI (Figure 2).

Subsequently, the patient underwent surgical drainage immediately because conservative treatment with antibiotics had been ineffective in the first treatment. The abscess cavity was located in the rectus abdominis muscle, a large amount of white pus was excreted, and no bacteria were detected. After drainage, the abscess disappeared promptly, and he was discharged after three weeks. He had no postoperative complications, but his discharge was postponed due to the adjustment of the oral dose of warfarin potassium.

## Discussion

PAWAs are extremely rare. Muscles have a lesser risk of infection due to abundant blood flow. Inflammation does not easily spread into the muscles, even with sepsis [1]. Most inflammatory diseases of the abdominal wall are caused by postoperative wound infection or the spread of intraperitoneal abscesses [2]. Therefore, there are only a few reports on PAWA (Table 1) [3-7].

**Table 1:** Review of previous reports of primary abdominal wall abscess Half of the patients with primary abdominal wall abscess have comorbidities. Cultured bacteria varied among patients. Most patients were successfully treated by surgical drainage. AF: atrial fibrillation; CRF: chronic renal failure; DM: diabetes mellitus; F: female; M: male.

Case	Author	Year	Age	Sex	Past history	Cultured bacteria	Treatment
1	Weiner et al. [3]	1975	54	F	DM	Streptococcus	Surgical drainage
2	Tamura et al. [4]	2002	40	M	Cirrhosis	Streptococcus	Surgical drainage (laparotomy)
3	Malhotra [5]	2012	62	M	None	Mycobacterium tuberculosis	Fine-needle suction and antitubercular drug
4	Mora-Guzmán et al. [6]	2017	45	M	None	Actinomyces, Eikenella corrodens	Conservative treatment
5	Shinohara et al. [7]	2019	20	M	None	Staphylococcus aureus	Surgical drainage
6	Our case	2021	81	M	DM, AF, CRF	Citrobacter koseri	Conservative treatment and surgical drainage

The mechanism of PAWA remains unclear. Previous reports have listed muscle damage, diabetes mellitus, and liver cirrhosis as risk factors for PAWA [3,4,7]. Compromised conditions and malnutrition, such as diabetes and liver cirrhosis, could relate to infection. Especially in patients with cirrhosis, abdominal collateral circulation is also associated with abdominal wall abscess [4]. Our case also had many comorbidities such as diabetes mellitus, chronic renal failure, and arrhythmia, which suggested susceptibility to infection. Our patient's postoperative pneumonia indicated that he was susceptible to infection and further recurrent abdominal abscesses. Some previous reports of primary iliopsoas abscesses showed bacterial infection in blood cultures, suggesting hematogenous infections of the primary muscle abscess [8,9]. According to previous reports, the causative bacteria of PAWA were identified in all cases, but different in each case. There are reports of PAWA in patients without any medical history [5,6]. We suggest that hematogenous infection in compromised conditions is strongly related to PAWA. Since the number of cases of PAWA is still small, the cause of its occurrence is unrevealed. It is necessary to accumulate PAWA cases.

Goodman et al. reported that images of abdominal wall abscesses are characterized by cellulitis and fluid collection, with gas in 30% [2]. However, it is difficult to diagnose abscesses, hematomas, and necrotic tumors by imaging alone [2,4]. Comprehensive diagnosis combining imaging, clinical findings, bacterial culture, and cytology is necessary.

The treatment of muscle abscesses is surgical drainage and antibiotics. Small abscesses could only be treated with antibiotics alone [5]. However, if the abscess grows and inflammation spreads to the abdominal organs, laparotomy is needed [6]. Thus, early drainage is essential. In previous reports, most cases (83.3%, 5/6 cases) were successfully treated by drainage. In our case, however, it took several days to diagnose PAWA. Antibiotic treatment was not effective at the first admission, and both abscesses improved promptly after surgical drainage. Compromised patients with many comorbidities tend to have exacerbated infections, so they require early drainage.

## Conclusions

A comprehensive diagnostic assessment, including bacterial culture and cytology, is needed for PAWA. Patients with PAWA are sometimes compromised, thus delay of treatment lead to exacerbated infection. For a case with an isolated abdominal wall abscess, PAWA should be considered as one of the diagnoses. Further, early drainage should be considered.
